# Risk of testicular cancer and exposure to welding fumes

**DOI:** 10.1093/eurpub/ckaf255

**Published:** 2026-01-10

**Authors:** Benjamin Kendzia, Thomas Behrens, Thomas Brüning, Andreas Stang, Karl-Heinz Jöckel, Wolfgang Ahrens

**Affiliations:** Institute for Prevention and Occupational Medicine of the German Social Accident Insurance, Institute of the Ruhr University Bochum (IPA), Bochum, Germany; Institute for Prevention and Occupational Medicine of the German Social Accident Insurance, Institute of the Ruhr University Bochum (IPA), Bochum, Germany; Institute for Prevention and Occupational Medicine of the German Social Accident Insurance, Institute of the Ruhr University Bochum (IPA), Bochum, Germany; Institute for Medical Informatics, Biometry, and Epidemiology (IMIBE), University Hospital Essen, Germany; School of Public Health, Bos, Boston University, Boston, MA, United States; Institute for Medical Informatics, Biometry, and Epidemiology (IMIBE), University Hospital Essen, Germany; Leibniz-Institute for Prevention Research and Epidemiology-BIPS GmbH and Institute for Statistics, University of Bremen, Epidemiological Methods and Etiological Research, Bremen, Germany

## Abstract

The incidence of germ-cell testicular cancer (TC) has increased in recent decades. Current evidence does not indicate a unanimous association between occupational exposure to welding fumes and TC risk. However, most publications do not provide information on exposure levels to welding fumes. We investigated the association between occupational exposure to welding fumes and the risk of TC in a German case–control study (268 cases and 797 control subjects). A measurement-based welding-exposure matrix was used to estimate year-specific exposure values which were linked to job-task descriptions. Odds ratios (ORs) with 95% confidence intervals (CIs) were calculated via logistic regression, conditional on study area and 5-year age groups. ORs were adjusted for a history of cryptorchidism, a family history of TC, and ever exposure to metal-working fluids (MWF). Regular welding was associated with an elevated TC risk (OR: 2.26, CI: 0.92–5.53) compared to occasional welding (OR: 1.14, CI: 0.78–1.69). For non-seminoma, there was an indication of a strong association among regular welders (OR: 3.71, CI: 1.08–12.80) and for non-welders using MWFs (OR: 3.39, CI: 1.21–9.53). However, these estimates were based on few exposed cases. We did not observe dose–effect relationships with increasing lifetime exposure to welding fumes, duration, or average intensity. We found an association between regular welding and TC risk based on standardized job-task descriptions, but not when using the year-specific exposure values.

## Introduction

Welding work is associated with various exposures that are hazardous to human health: Welding fumes are classified as human carcinogens by the International Agency for Research on Cancer [1]. Welding is associated with an increased risk for lung cancer. There is also some evidence connecting welding to rare cancers like small intestine and male breast tumors [2]. Despite a relatively low overall incidence, germ-cell testicular cancer (TC) is the most common cancer among young men aged 15–40 years [3]. The age-standardized incidence rate has remained almost constant recently, after having risen steadily for decades [4]. However, the reasons for this increase are unknown and risk factors for the disease are still poorly understood. A family history of TC by a first-degree relative [5] and a history of cryptorchidism can be considered as established though [6]. Several studies have suggested that exposures during adolescence or early life, including environmental factors such as pesticides [7] and diet [8], may be associated with the development of TC [9]. The theory of prenatal origins of TC is widely accepted [10].

There is only limited evidence regarding an association between occupational exposures and TC risk [7, 11, 12]. Increased risks were found among metalworkers, which were most pronounced in metal-cutting activities [13]. Long-term dermal exposure to metalworking fluids (MWFs) in the automobile industry was also a risk factor for the development of the non-seminoma subtype of TC [14].

Given the natural history of this cancer and the relatively young age at diagnosis, occupational exposures in certain time windows could be part of the initial stage of carcinogenic transformation [15]. Parental work in manufacturing or metal processing before birth is linked to higher TC risk in offspring [16, 17]. This would have strong implications for the prevention of a cancer that could originate prenatally [18]. For exposure to welding activities, weak and inconsistent associations were observed so far [19–21]. In these studies, exposure to welding fumes was assessed based on job titles or industries which does not provide information on the actual level of exposure. Job titles cannot characterize exposure to welding fumes specifically enough, as there is a great variability in exposure levels [22]. Different welding processes and materials generate different mixtures of particulate matter and carcinogens in welding fumes. The studies published so far were not consistent and evidence for determining for a possible association for TC among welders remains incomplete.

Therefore, we report TC risk estimates from a German case–control study, assessing lifetime welding fume exposure by detailed job histories linked to a welding exposure matrix based on a large number of personal measurements of inhalable welding fumes.

## Methods

### Study subjects

We estimated the association of TC risks and occupational welding using data of a population-based incident case–control study. Detailed descriptions of this study have been presented elsewhere [23]. In brief, eligibility criteria for cases included a diagnosis of TC between 1995 and 1997 with participants aged 15–64. Detailed interviews covered any welding-related job activities, sociodemographic characteristics, alcohol and tobacco consumption, and medical history across five German regions (cities of Bremen, Essen, Hamburg, Saarbrucken, and the Federal State of Saarland).

Cases were ascertained through an active reporting system from clinical and pathological departments. A reference pathologist independently assessed the cancer diagnosis by reviewing clinical data, histologic conclusions of the local pathologists, and by re-examining available tissue specimens, including slides or tissue blocks. A particular issue is the case of spermatocytic TC which appears to be a separate cancer entity with markedly different features from other germ cell tumors [24]. For this reason, one spermatocytic TC case was excluded. Interviews were completed with 268 of 353 eligible cases (76% response rate).

Control subjects were retrieved from the International Rare Cancer Study (Kendzia et al., 2022) and consisted of individuals without a cancer diagnosis, randomly selected from resident registries. They were frequency-matched based on region, sex, and 5-year age groups. Of 1982 eligible controls, 918 were interviewed. The response was 57% after removing controls that moved away or died before first contact. The Rare Cancer Study included seven cancer sites and aimed at recruiting a number of control subjects equaling four times the number of the most common cancer site in each matching stratum. Consequently, the number of control subjects was determined by the most frequent cancer within the respective stratum, and these controls were used in the present analysis. For this analysis, 121 control interviews were excluded because no matched cases were identified in the corresponding strata, leaving 797 control subjects for matching. For cases younger than 35 years, up to two control subjects were frequency-matched by 5-year group (15–19, 20–24, …, 30–34 years) and study region. The higher matching ratio in the older age group above 35 years was chosen to compensate at least to some extent for the loss of power because of lower case numbers. Due to the other cancer sites of the International Rare Cancer Study, the matching ratios in the higher age groups are in some cases much higher than 1:4.

### Exposure assessment

The job-related interview was conducted by trained interviewers. It started with the assessment of the lifelong work history including start and end year of each particular job held for six months or longer. Job-title information was coded according to the International Classification of Occupations, 1968 version (ISCO) [25]. Subsequently, a list of specific job tasks was presented to study participants which included a specific question on whether the study subject had ever performed welding tasks during one of his jobs. If the answer was yes, this question triggered the use of the welding-specific job-task questionnaire independent of the job history. If a study participant reported having ever welded in at least one job, a standardized job-specific welding questionnaire (JSQ) was used, soliciting detailed information on the applied welding process, materials used, and work environment. This JSQ contained information about frequencies of the individual welding processes (daily, several days a week, several days a month, less frequently) and the used base material. If a worker reported daily welding for at least six months in the JSQ or if at least one work period of at least six months in the general job history was coded as a regular welder (ISCO code 872**), the subject was classified as regular welder. A worker who was never classified as a regular welder but who reported to have welded as part of a different job, was classified as occasional welder.

Another JSQ dealt with metal-cutting activities and exposure to MWF. Subjects were asked whether they had carried out metal-cutting activities (drilling, molding or grinding and polishing of metals), whether they had used aqueous or oil-based MWFs and whether regular skin contact occurred during use. Study participants who reported exposure to MWF were defined as subjects who were ever exposed to MWFs. Workers who ever worked in an occupation with an ISCO code beginning with digits 7, 8, or 9 were classified as blue-collar workers. All subjects who reported not having welded served as reference group.

We used a welding-exposure matrix (WEM) with estimates from 15 473 inhalable airborne measurements taken at welding workplaces in Germany (1985–2016) [22]. Cumulative exposure to welding fumes was estimated by linking the WEM to welding-related activities reported in the JSQ. The welding processes reported in this supplementary questionnaire were classified according to the definitions used in the WEM (Supplementary Table S1).

Exposure assessment for each exposed subject and welding year followed a stepwise procedure as described recently [2, 26]: First, the shift concentrations of the WEM (μg/m^3^) were linked with the welding tasks of the participants. Exposed subjects who reported that they simultaneously performed several welding tasks were additionally weighted by the time of each welding activity. Second, values were multiplied by the factor for the welded material according to Kendzia et al. [22] (mild steel = 1, stainless steel = 0.61, aluminum = 1.06, other or mixed content = 0.84). Here, welding of stainless steel was associated with 0.61-fold lower concentrations as compared to mild steel. Third, values were multiplied by a frequency factor for welding activities (daily = 1, several days/week = 0.5, several days/month = 0.2, less frequently = 0.1). Afterwards, values were multiplied by the factor of daily intensity of welding according to the median welding hours in a German case–control study (HdA study) on lung cancer [26]. Full-shift exposure was assumed for regular welding; otherwise, 3 hours for occasional welding. The sum of this product of the individual welding-years resulted in the cumulative welding fumes exposure (μg/m^3^-years).

### Statistical analysis

To investigate the association between welding and TC, conditional logistic regression models were calculated to estimate odds ratios (ORs) and 95% confidence intervals (CIs). First, we estimated crude OR estimates (OR1) without adjustments. Matching factors for the conditional analyses were study area and 5-year age group (OR2 and OR3). OR3 was further adjusted for a history of cryptorchidism, a family (fathers and/or brothers) history of TC, and for ever exposure to MWFs. Interactions on an additive scale between ever exposed to welding fumes and MWFs were assessed by fitting linear OR models and calculating the relative excess risk due to interaction (RERI) with likelihood-based CI estimates in order to estimate the departure from additivity of the effects of both possible risk factors combined [27]. Pairwise joint effects between these factors were estimated as OR3 but without adjustment for exposure to MWFs (OR4).

Cumulative lifetime exposure, duration (defined as the sum of exposure duration across different job periods), average intensity, and time since last welding were categorized by median and tertiles of exposed controls, respectively. Trend tests for the possible exposure-response relationships used logistic regression with log-transformed exposures for better fit. Analyses were stratified by seminoma and non-seminoma tumor types [28].

The robustness of OR estimates was examined by additional adjustment for adult height as suspected risk factor [29]. Because Saarbrucken was the smallest study area and is also a city in the federal state of Saarland, we also estimated risks considering Saarland and Saarbrucken as one study region. We also restricted the analysis group to blue-collar workers to limit residual confounding from socio-economic factors. Men aged 50+ were excluded in sensitivity analyses to remove possible spermatocytic TC cases. Analyses were performed using SAS, version 9.4 (SAS Institute, Inc., Cary, NC, USA).

## Results

Table 1 presents characteristics of 268 TC cases and 797 control subjects. The case group consisted of 169 seminomas and 99 non-seminomas. The proportion of exposed men who were exposed to welding fumes was very similar between the controls (31%) and the cases (32%). Overall, cases with seminoma were older than cases with non-seminoma. A higher proportion of cases had a history of cryptorchidism.

**Table 1.** Characteristics of the Study Population by Disease Status, Germany, 1995–1997

		Controls		All cases		Seminoma		Non-seminoma	
Characteristic	Category	No.	%	No.	%	No.	%	No.	%
N of male participants		797		268		169		99	
Study area	Bremen	302	38	125	47	82	49	43	43
	Essen	129	16	56	21	26	15	30	30
	Hamburg	69	9	25	9	22	13	3	3
	Saarbrucken	12	2	6	2	3	2	3	3
	Saarland	285	36	56	21	36	21	20	20
Ever exposed to welding fumes	Never	546	69	181	68	114	68	67	68
	Ever	251	31	87	32	55	32	32	32
	Occasional welding	234	29	77	29	50	29	27	27
	Regular welding	17	2	10	4	5	3	5	5
Age at diagnosis/interview, years	15–24	67	8	25	27	4	2	21	21
	25–34	301	38	116	28	69	41	47	47
	35–44	235	29	96	29	72	42	24	24
	45–54	83	10	19	19	15	9	4	4
	55–64	111	14	12	5	9	5	3	3
History of cryptorchidism	Yes	27	3	39	15	25	15	14	14
	No	755	95	225	84	140	83	85	86
	Unknown	15	2	4	1	4	2	0	0
Family history of TC	Yes	6	1	11	4	7	4	4	4
	No	791	99	257	96	162	96	95	96
Ever exposed to MWF	Yes	165	21	55	21	34	20	21	22
	No	626	78	212	79	135	80	77	78
	Unknown	6	1	1	0	0	0	1	0

a Abbreviations: MWF, metal-working fluids; TC, testicular cancer.

Table 2 shows the OR estimates for TC associated with welding-related occupations. For ever welding, the risks for TC were slightly increased. Stratification of exposed men into regular and occasional welders revealed higher adjusted risk estimates for regular (OR3: 2.26, CI: 0.92–5.53) than for occasional welders (OR3: 1.14, CI: 0.78–1.69). The highest adjusted ORs associated with regular welders were found for the non-seminoma subtype (OR3: 3.71, CI: 1.08–12.80). For several major welding processes slightly increased risks of TC were suggested (autogenous welding OR3: 1.39, CI: 0.89–2.19; torch cutting OR3: 1.22, CI: 0.71–2.11; gas metal arc welding OR3: 1.20, CI: 0.68–2.12). We did not observe a clear trend relative to time since last welding-fume exposure (OR3: 1.00, CI: 0.86–1.18), ranging from an OR3 for current welding of 1.22 (CI: 0.71–2.09) to a relative risk after ≥17 years of welding of 1.08 (CI: 0.52–2.21). Among current welders the risk was highest for seminoma (OR3: 1.53, CI: 0.83–2.82), but lowest for the non-seminoma subtype (OR3: 0.66, CI: 0.25–1.73).

**Table 2.** Testicular-cancer risks among workers performing welding tasks by type of welding process, time since last welding, and by tumor histology, Germany, 1995–1997

	Controls	All cases						Seminoma			Non-seminoma		
Welding activity	No.	No.	OR1^a^	OR2^b^	95% CI	OR3^c^	95% CI	No.	OR3^c^	95% CI	No.	OR3^c^	95% CI
Reference group^d^	546	181	1.00	1.00		1.00		114	1.00		67	1.00	
Ever welding	251	87	1.05	1.14	0.84–1.56	1.21	0.83–1.77	55	1.18	0.75–1.86	32	1.23	0.67–2.22
Occasional welding	234	77	0.99	1.09	0.79–1.50	1.14	0.78–1.69	50	1.15	0.72–1.83	27	1.11	0.60–2.04
Regular welding	17	10	1.78	1.91	0.82–4.44	2.26	0.92–5.53	5	1.60	0.53–4.84	5	3.71	1.08–12.80
Applied process													
Autogenous welding	181	65	1.08	1.21	0.86–1.72	1.39	0.89–2.19	39	1.39	0.80–2.40	26	1.36	0.67–2.75
Torch cutting	133	44	1.00	1.16	0.78–1.74	1.22	0.71–2.11	28	1.36	0.71–2.61	16	0.92	0.36–2.37
Spot welding	96	39	1.22	1.32	0.86–2.03	1.11	0.65–1.89	22	0.99	0.51–1.89	17	1.20	0.53–2.70
MMAW	171	55	0.97	1.09	0.76–1.57	1.10	0.68–1.79	35	1.19	0.67–2.10	20	0.86	0.38–1.95
TIGW	28	15	1.62	1.52	0.77–3.01	0.88	0.37–2.09	8	0.74	0.26–2.07	7	1.13	0.31–4.12
GMAW	97	39	1.21	1.29	0.84–1.99	1.20	0.68–2.12	24	1.34	0.69–2.62	15	0.89	0.33–2.35
TSL welding, years^e^			0.95	1.01	0.87–1.16	1.00	0.86–1.18		0.94	0.77–1.14		1.12	0.88–1.43
Current welding (0)	85	36	1.28	1.29	0.83–2.01	1.22	0.71–2.09	26	1.53	0.83–2.82	10	0.66	0.25–1.73
Q1 and Q2 (1–12)	83	27	0.98	0.95	0.59–1.54	0.94	0.54–1.65	14	0.79	0.42–1.50	13	1.21	0.61–2.39
Q3 and Q4 (13–48)	83	24	0.87	1.17	0.69–2.00	1.21	0.67–2.17	15	0.78	0.38–1.60	9	1.18	0.53–2.60
T1 (1–7)	51	15	0.89	0.99	0.53–1.84	0.96	0.48–1.89	6	0.65	0.25–1.67	9	1.33	0.55–3.24
T2 (8–16)	53	22	1.25	1.12	0.65–1.93	1.13	0.61–2.09	14	1.05	0.49–2.22	8	1.26	0.50–3.18
T3 (17–48)	62	14	0.68	1.00	0.51–1.95	1.08	0.52–2.21	9	1.00	0.42–2.40	5	1.27	0.41–3.92

Abbreviations: CI, confidence interval; GMAW, Gas metal arc welding; MMAW, Manual metal arc welding; OR, odds ratio; Q, quartile; T, tertile; TIGW, Tungsten inert gas welding; TSL welding, Time since last welding in years.

a Crude OR without any adjustment.

b OR conditional on study area and 5-year age group.

c OR additional adjusted for specific risk factors: history of cryptorchidism, suffering from testicular cancer of the father/brother, and ever exposure to metal-working fluids.

d Subjects who were never exposed to welding fumes.

e OR were computed by entering the log-transformed continuous variable (time since last welding +1) into the model.

Table 3 shows the risk estimates for the exposure-effect relationships welding-fume exposure and TC risk. For cumulative exposure above the median of the exposed controls (831 µg/m^3^-years), an increased risk was observed (OR3: 1.30, CI: 0.80–2.13). With increasing cumulative exposure to welding fumes, the risks of developing TC (OR3: 1.03, CI: 0.97–1.09), seminoma (OR3: 1.03, CI: 0.96–1.10), and non-seminoma subtype (OR3:1.03, CI: 0.96–1.10) were not elevated. In the highest exposure tertile of cumulative exposure, we did not observe an increased OR for TC (OR3: 1.04, CI: 0.58–1.87). There was no clear tendency for an increased risk of TC with increasing duration of exposure (overall OR3: 1.10, CI: 0.94–1.28; seminoma OR3:1.11, CI: 0.92–1.32; non-seminoma OR3: 1.06, CI: 0.82–1.38) or average intensity (overall OR3: 0.98, CI: 0.71–1.36; seminoma OR3: 1.01, CI: 0.70–1.48; non-seminoma OR3: 0.97, CI: 0.59–1.61), although increased ORs were observed for the second tertile.

**Table 3.** Testicular-cancer risks among workers performing welding tasks by cumulative fume exposure, duration, and average intensity of welding fume exposure by tumor histology, Germany, 1995–1997

	Controls	All cases						Seminoma			Non-seminoma		
Welding activity	No.	No.	OR1^a^	OR2^b^	95% CI	OR3^c^	95% CI	No.	OR3^c^	95% CI	No.	OR3^c^	95% CI
Reference group^d^	546	181	1.00	1.00		1.00		114	1.00		67	1.00	
Cumulative exposure^e^^,^^f^			1.00	1.02	0.97–1.06	1.03	0.97–1.09		1.03	0.96–1.10		1.03	0.94–1.13
Q1 and Q2 (0–830)	125	46	1.10	1.15	0.78–1.71	1.16	0.75–1.79	28	1.08	0.64–1.84	18	1.21	0.63–2.32
Q3 and Q4 (831–39 704)	126	41	0.98	1.13	0.75–1.71	1.30	0.80–2.13	27	1.34	0.75–2.41	14	1.26	0.56–2.82
T1 (0–431)	81	34	1.27	1.32	0.84–2.07	1.24	0.75–2.04	21	1.21	0.67–2.20	13	1.22	0.59–2.54
T2 (432–1456)	84	30	1.08	1.16	0.72–1.86	1.32	0.78–2.23	19	1.27	0.67–2.39	11	1.32	0.58–3.00
T3 (1457–39 704)	86	23	0.81	0.94	0.56–1.56	1.04	0.58–1.87	15	1.04	0.52–2.08	8	1.09	0.42–2.79
Duration of welding^e^ years			1.01	1.06	0.93–1.21	1.10	0.94–1.28		1.11	0.92–1.32		1.06	0.82–1.38
Q1 and Q2 (0–8)	125	46	1.11	1.21	0.81–1.79	1.31	0.83–2.07	29	1.35	0.78–2.34	17	1.20	0.60–2.41
Q3 and Q4 (9–49)	126	41	0.98	1.08	0.72–1.62	1.12	0.70–1.79	26	1.04	0.59–1.81	15	1.26	0.60–2.61
T1 (1–4)	75	21	0.85	0.95	0.56–1.62	1.04	0.59–1.86	11	0.88	0.42–1.85	10	1.19	0.52–2.72
T2 (5–12)	86	39	1.37	1.35	0.88–2.07	1.40	0.86–2.30	24	1.38	0.76–2.50	15	1.42	0.69–2.95
T3 (13–49)	90	27	0.91	1.08	0.66–1.75	1.16	0.67–2.00	20	1.25	0.67–2.31	7	0.93	0.35–2.47
Average intensity^e^^,^^g^			0.83	0.83	0.62–1.12	0.98	0.71–1.36		1.01	0.70–1.48		0.97	0.59–1.61
Q1 and Q2 (0–92)	125	47	1.13	1.25	0.84–1.84	1.25	0.81–1.94	28	1.12	0.66–1.91	19	1.37	0.71–2.63
Q3 and Q4 (93–1038)	126	40	0.96	1.04	0.69–1.57	1.16	0.71–1.90	27	1.27	0.71–2.26	13	1.00	0.45–2.23
T1 (0–64)	80	30	1.13	1.19	0.74–1.90	1.15	0.69–1.91	20	1.13	0.62–2.06	10	1.09	0.49–2.38
T2 (65–142)	85	34	1.21	1.31	0.84–2.06	1.40	0.83–2.35	20	1.31	0.69–2.48	14	1.49	0.68–3.28
T3 (143–1038)	86	23	0.81	0.92	0.55–1.53	1.10	0.62–1.95	15	1.13	0.57–2.25	8	1.14	0.46–2.83

Abbreviations: CI, confidence interval; OR, odds ratio Q, quartile; T, tertile.

a Crude OR without any adjustment.

b OR conditional on study area and 5-year age group.

c OR additional adjusted for specific risk factors: History of cryptorchidism, suffering from testicular cancer of the father/brother, and ever exposure to metal-working fluids.

d Subjects who were never exposed-welding fumes.

e OR were computed by entering the log-transformed continuous variable (cumulative exposure + 1; duration + 1; average intensity + 1) into the model.

f Cumulative exposure to welding fumes presented in μg/m^3^-years.

g Average intensity of exposure to welding fumes presented in μg/m^3^.

Both pairwise joint exposures of welding and MWFs were associated with an increased risk of TC when compared to the never exposed reference group (for welding: OR4: 1.55, CI: 1.02–2.34; for MWFs: OR4: 2.13, CI: 1.05–4.33) (Table 4). For the non-seminoma subtype in particular, the risk estimate was highest for men using MWFs without welding activities (OR4: 3.39, CI: 1.21–9.53), based on six exposed non-seminoma cases. The joint effect on the risk of TC (RERI: −0.66, CI: −2.09 to 0.29) and of both subtypes was below additivity.

**Table 4.** Individual and joint effects (testicular-cancer risks) and relative excess risk due to interaction in relation to welding and meta-working-fluids exposure by tumor histology, Germany, 1995–1997

Exposure Metric		Controls	All cases						Seminoma			Non-seminoma		
Ever welding	Ever MWF	No.	No.	OR1^a^	OR2^b^	95% CI	OR4^c^	95% CI	No.	OR4^c^	95% CI	No.	OR4^c^	95% CI
No	No	511	167	1.00	1.00		1.00		104	1.00		59	1.00	
Yes	No	115	46	1.22	1.47	0.98–2.20	1.55	1.02–2.34	29	1.39	0.85–2.27	17	1.83	0.98–3.40
No	Yes	32	14	1.34	2.10	1.04–4.23	2.13	1.05–4.33	8	1.69	0.71–4.01	6	3.39	1.21–9.53
Yes	Yes	133	41	0.94	1.00	0.67–1.50	1.01	0.66–1.53	26	0.99	0.60–1.63	15	0.97	0.51–1.86
RERI^d^							−0.66	−2.09 to 0.29		−0.95	−3.34 to 0.44		−0.53	−2.23 to 0.55

Abbreviations: CI, confidence interval; MWF, metal-working fluids; OR, odds ratio; RERI, relative excess risk due to interaction.

a Crude OR without any adjustment.

b OR conditional on study area and 5-year age group.

c OR additional adjusted for history of cryptorchidism and suffering from testicular cancer of the father/brother.

d RERI resulting from the linear OR model with likelihood-based 95% CI derived via 1000 bootstrap replications of the original data.

Restricting the study population to blue-collar workers slightly attenuated the risk estimates (Supplementary Table S2). Considering Saarbrucken and the Saarland as one study area, also led to slightly decreased risk estimates (data not shown). Limiting the analysis to subjects below age 50 years at the time of diagnosis or interview yielded similar results (Supplementary Table S3).

## Discussion

In this analysis, no consistent dose–effect relationships were found for welding fumes exposure, duration, or intensity, but regular welder had higher TC risk. Men exposed to MWFs without welding activities showed increased non-seminoma risk, though based on few cases so that both effects showed wide confidence intervals. Joint effects of welding and MWFs were below additive. Current welders had higher risk for seminomas than for non-seminomas.

One major advantage of this study over previous analyses is that lifetime exposure to welding fumes was assessed based on detailed and standardized welding-related job task questionnaire modules and the quantification of welding-fume exposure by linking specific job tasks to a previously published WEM [22]. The WEM is based on a high number of personal measurements of inhalable welding fumes at different welding workplaces in Germany. It was possible to use each exposed subject’s complete professional welding history, considering exposure probability, frequency, intensity, type of welding process, and duration for each welding-job period. Therefore, we were able to include the high variability of exposure in our exposure assessment, both between and within exposed subjects, due to the different working conditions and the different types of welding. Job periods were coded by experienced personnel and coding was blinded to case–control status.

Risks were adjusted for cryptorchidism, family history, and MWF use, with little effect except for tungsten inert gas welding. Adjusting for height [29] did not change our results (data not shown). The pathological samples were examined by the most experienced pathologist with specific expertise in TC at the time. Thus, the case ascertainment and pathological classification were the best available at its time. Spermatocytic TC show different histologic features than other germ cell tumors and used to be classified as seminomas in the past. They occur later in life (> 50 years) and may share a completely different etiology than other testicular germ cell tumors [30]. We conducted a sensitivity analysis, restricting to subjects <50 years of age (18 men), which did not affect results substantially. As a further sensitivity analysis, we also restricted the analyses to blue‑collar workers to minimize potential residual confounding by socioeconomic position. This is particularly relevant given that TC have historically been associated with higher socioeconomic status [31], whereas increased mortality has been linked to lower socioeconomic status [32]. The sensitivity analysis produced results consistent with the main findings, supporting the robustness of our conclusions.

A limitation of this study is the small number of welding-exposed cases, especially for non-seminoma allowing for the possibility of a chance finding. The focus of this study was to assess the impact of lifetime exposure to welding fumes on TC, but other exposures at welding workplaces like MWFs, electromagnetic fields, hexavalent chromium or nickel may affect TC risk. Further, MWFs are used in machining processes to cool and lubricate a workpiece and include oil-water emulsions, pastes, gels or oil-based chemicals, which may also cause occupational diseases [33]. In a case–control study among automobile workers, an association was found between the use of MWFs and non-seminoma TC [14]. In line with these results, we also observed an increased risk, particularly for the non-seminomas. All risk estimates were much higher for men who had used MWFs without welding activities than for men who welded but were not exposed to MWFs. The joint effect on TC risk and of both subtypes was below additivity. Given the small number of exposed subjects and the complex, heterogeneous nature of welding fumes and MWFs, it is difficult to identify or isolate any specific mechanisms that might explain this observation.

Exposure misclassification may bias results in case–control studies. The assessment of a lifelong occupational history is presumable highly specific. Furthermore, we used detailed job-specific questionnaires as well as measurement data from German workplaces, implying that the level of welding-fume exposure for comparable welding settings was the same and, therefore, all subjects performing the same tasks are assigned the same average exposure value. These restrictions may have led to incorrect estimates of welding-fume exposure, which may lead to under- or overestimation of risks, especially when continuous data are categorized [2, 34].

In our study, median welding exposure in controls was low at 831 μg/m^3^-years, lower than in a lung-cancer study among older welders with 1838 μg/m^3^-years [26] and also lower than in previous analysis of risks of various rare cancers (1586 μg/m^3^-years [2]), using the same WEM as in this analysis. Here, autogenous welding, a low-emission process, was most common. TC mainly affects young men with lower lifetime exposure and shorter time-lag between exposure and diagnosis, possibly explaining increased risk in current welders but no clear dose–effect relationship. Because we focused on welding-fume exposure and did not solicit other exposures associated with welding activities, these exposures may also play a role.

Few studies that investigated the association between welding activities and TC have been published so far with inconsistent findings. A recent French case–control study (2015–2018), found increased TC risk among regular welders and flame-cutters (OR: 1.40, CI: 0.59–3.29) [19]. Comparable to our results, the authors observed a higher risk for non-seminoma (OR: 2.41, CI: 0.74–7.81) than for seminoma TC (OR: 1.16, CI: 0.40–3.36). A number of studies investigated whether there are differences in risk factors between the seminoma and non-seminoma subtype [35]. Non-seminomas are rarer, more aggressive, diagnosed earlier than seminomas and often include multiple cell types [36]. It is s debated if they are distinct diseases or stages of the same [37]. It has been suggested that non-seminoma develops through the reprogramming of a seminoma tumor cell into a pluripotent embryonal carcinoma cell, which should be considered as a disease entity at different stages of development [38]. The risk factors should therefore not differ between seminoma and non-seminoma. Accordingly, a systematic review found no clear difference in terms of medical, lifestyle, socioeconomic, pregnancy-related, or genetic risk-factors between the two subtypes [39].

A French hospital-based study including 20 exposed cases found a similar TC risk for occasional welders (OR: 1.49, CI: 0.53–4.15) [23], while a Danish cohort of 10 059 metal workers showed no increased risk [21]. The lack of association with exposure in adulthood is in line with the prevailing research hypothesis that supports a prenatal origin of TC. This supports the idea that TC likely originates prenatally, with occupational exposures possibly promoting existing tumors [7, 35]. In view of the discovery of carcinomas *in situ* in boys before puberty, it is assumed that the cancer could be activated by an initiation event *in utero* or in infancy. For this reason, studies were analysed that focused on the occupational exposure of parents of sons diagnosed with TC. A registry-based case–control study conducted in the Nordic countries evaluated paternal/maternal welding-fumes exposure and TC risk, provided some evidence of associations in offspring, with no clear dose–effect relationship [17]. No evidence of a positive association between paternal welding-fumes exposure was found in a French study of TCs in sons [40].

In summary, there was no clear evidence of an association between three exposure metrics (cumulative, duration, and intensity) of welding fumes and TC. Regular welders showed higher risk, mainly for non-seminoma, but estimates lacked precision. Stronger risks, albeit again based on small numbers, were seen with MWF exposure. However, the overall impact of welding-fumes exposure on TC risk appears modest. Exposures other than welding fumes, which are prevalent in the job history of metal workers who—amongst others—performed welding tasks, might explain the observed inconsistent dose–effect relationship.

## Supplementary Material

ckaf255_Supplementary_Data

## Data Availability

The data are not publicly available due to containing information that could compromise the privacy of the study participants. Key points Regular welders showed increased risks of testicular cancer for the non-seminoma subtype. Exposure to metalworking fluids was associated with increased risks. We did not find a clear association between welding-fume exposure (cumulative, duration, intensity) and testicular cancer risk. Other workplace exposures of metalworkers may explain the inconsistent findings associated with welding-fume exposure. Our results provide further evidence for health and safety authorities to reduce exposure to welding fumes.
