# Supplementary material for: Risk of testicular cancer and exposure to welding fumes
Source: Eur J Public Health. 2026 Jan 10;36(2):ckaf255. doi: 10.1093/eurpub/ckaf255 (PMC13064841; doi:10.1093/eurpub/ckaf255)
Supplement: ckaf255_Supplementary_Data [file ckaf255_supplementary_data.zip › Supplement_Data.docx]

**Supplementary Table 1.** Welding Exposure Matrix (WEM) with Estimates of Average Shift Concentrations of Welding Fume for Major Welding Processes according Kendzia et al. 2019

| **Welding process** | **Welding fume [µg/m^3^]** |
| --- | --- |
| Gas welding | 900 |
| Autogenous welding | 900 |
| Torch cutting | 2,800 |
| Spot welding | 500 |
| Manual metal arc welding | 2,300 |
| Tungsten inert gas welding | 700 |
| Gas metal arc welding | 3,200 |
| Other or mixed welding process | 1,700 |

**Supplementary Table 2.** Testicular-Cancer Risks among Workers in Welding-Related Occupations for Cumulative, Duration, and Average Intensity of Exposure by Tumor Histology, Germany, 1995-1997. Sensitivity analysis restricted to blue-collar workers.

|  | **Controls** | **All cases** | | | | | | **Seminomas** | | | **Non-seminomas** | | |
| --- | --- | --- | --- | --- | --- | --- | --- | --- | --- | --- | --- | --- | --- |
| Welding fumes metric | No. | No. | OR1^b^ | OR2^c^ | 95% CI | OR3^d^ | 95% CI | No. | OR3^d^ | 95% CI | No. | OR3^d^ | 95% CI |
| Reference group^a^ | 359 | 119 | 1.00 | 1.00 |  | 1.00 | Referent | 76 | 1.00 |  | 43 | 1.00 |  |
|  |  |  |  |  |  |  |  |  |  |  |  |  |  |
| Ever welding | 251 | 87 | 1.05 | 1.09 | 0.78-1.52 | 1.18 | 0.79-1.75 | 55 | 1.19 | 0.74-1.92 | 32 | 1.12 | 0.60-2.08 |
| Occasional | 234 | 77 | 0.99 | 1.03 | 0.73-1.46 | 1.11 | 0.74-1.67 | 50 | 1.15 | 0.71-1.88 | 27 | 1.01 | 0.53-1.91 |
| Regular | 17 | 10 | 1.78 | 1.79 | 0.76-4.22 | 2.16 | 0.87-5.39 | 5 | 1.65 | 0.54-5.03 | 5 | 3.28 | 0.92-11.7 |
|  |  |  |  |  |  |  |  |  |  |  |  |  |  |
| Cumulative exposure^e,f^ |  |  |  | 1.01 | 0.96-1.06 | 1.02 | 0.97-1.09 |  | 1.03 | 0.96-1.10 |  | 1.01 | 0.92-1.11 |
| Q1 and Q2 (0-830) | 125 | 46 | 1.10 | 1.10 | 0.73-1.68 | 1.14 | 0.72-1.80 | 28 | 1.10 | 0.63-2.46 | 18 | 1.14 | 0.58-2.26 |
| Q3 and Q4 (831-39,704) | 126 | 41 | 0.98 | 1.07 | 0.70-1.64 | 1.23 | 0.74-2.05 | 27 | 1.22 | 0.73-2.04 | 14 | 1.07 | 0.47-2.42 |
| T1 (0-431) | 81 | 34 | 1.27 | 1.25 | 0.78-2.00 | 1.22 | 0.73-2.04 | 21 | 1.25 | 0.68-2.30 | 13 | 1.14 | 0.53-2.42 |
| T2 (432-1,456) | 84 | 30 | 1.08 | 1.13 | 0.69-1.85 | 1.29 | 0.75-2.23 | 19 | 1.27 | 0.66-2.46 | 11 | 1.22 | 0.52-2.84 |
| T3 (1,457-39,704) | 86 | 23 | 0.81 | 0.88 | 0.52-1.49 | 0.98 | 0.54-1.78 | 15 | 1.02 | 0.50-2.07 | 8 | 0.93 | 0.36-2.42 |
|  |  |  |  |  |  |  |  |  |  |  |  |  |  |
| Duration of welding^e^. years |  |  |  | 1.05 | 0.91-1.20 | 1.09 | 0.93-1.29 |  | 1.12 | 0.93-1.35 |  | 1.03 | 0.78-1.34 |
| Q1 and Q2 (0-8) | 125 | 46 | 1.10 | 1.12 | 0.74-1.71 | 1.25 | 0.78-2.00 | 29 | 1.32 | 0.75-2.32 | 17 | 1.09 | 0.53-2.25 |
| Q3 and Q4 (9-49) | 126 | 41 | 0.98 | 1.05 | 0.69-1.62 | 1.10 | 0.68-1.79 | 26 | 1.07 | 0.60-1.91 | 15 | 1.14 | 0.54-2.44 |
| T1 (1-4) | 75 | 21 | 0.85 | 0.86 | 0.49-1.50 | 0.98 | 0.54-1.77 | 11 | 0.88 | 0.41-1.86 | 10 | 1.03 | 0.44-2.42 |
| T2 (5-12) | 86 | 39 | 1.37 | 1.30 | 0.83-2.04 | 1.38 | 0.83-2.29 | 24 | 1.39 | 0.75-2.58 | 15 | 1.34 | 0.63-2.83 |
| T3 (13-49) | 90 | 27 | 0.91 | 1.05 | 0.63-1.73 | 1.14 | 0.65-1.99 | 20 | 1.26 | 0.66-2.39 | 7 | 0.86 | 0.32-2.32 |
|  |  |  |  |  |  |  |  |  |  |  |  |  |  |
| Average intensity^e,g^ |  |  |  | 0.83 | 0.62-1.12 | 0.98 | 0.71-1.36 |  | 1.01 | 0.70-1.48 |  | 0.97 | 0.59-1.61 |
| Q1 and Q2 (0-92) | 125 | 47 | 1.13 | 1.20 | 0.79-1.81 | 1.24 | 0.78-1.94 | 28 | 1.15 | 0.66-2.00 | 19 | 1.29 | 0.65-2.53 |
| Q3 and Q4 (93-1,038) | 126 | 40 | 0.96 | 0.98 | 0.64-1.51 | 1.10 | 0.67-1.82 | 27 | 1.24 | 0.68-2.25 | 13 | 0.87 | 0.39-1.98 |
| T1 (0-64) | 80 | 30 | 1.13 | 1.19 | 0.73-1.94 | 1.18 | 0.70-1.99 | 20 | 1.19 | 0.64-2.21 | 10 | 1.06 | 0.48-2.37 |
| T2 (65-142) | 85 | 34 | 1.21 | 1.23 | 0.77-1.97 | 1.33 | 0.78-2.27 | 20 | 1.29 | 0.67-2.50 | 14 | 1.31 | 0.58-2.97 |
| T3 (143-1,038) | 86 | 23 | 0.81 | 0.85 | 0.50-1.44 | 1.10 | 0.56-1.82 | 15 | 1.07 | 0.53-2.16 | 8 | 0.97 | 0.39-2.46 |

Abbreviations: CI, confidence interval; OR, odds ratio Q, quartile; T, tertile.
a: Subjects who were never exposed-welding fumes.
b: Crude OR without any adjustment.
c: OR conditional on study area and 5-year age group.
d: OR additional adjusted for specific risk factors: History of cryptorchidism, suffering from testicular cancer of the father / brother and and ever exposure to metal-working fluids.
e: OR were computed by entering the log-transformed continuous variable (cumulative exposure+1; duration+1; average intensity+1) into the model.
f: Cumulative exposure to welding fumes presented in μg/m^3^-years.
g: Average intensity of exposure to welding fumes presented in μg/m^3^.

**Supplementary Table 3.** Testicular-Cancer Risks among Workers Performing Welding Tasks by Cumulative Fume Exposure, Duration, and Average Intensity of Fume Exposure by Tumor Histology, Germany, 1995-1997. Sensitivity analysis restricted to subjects < 50 years of age.

|  | **Controls** | **All cases** | | | | | | **Seminomas** | | | **Non-seminomas** | | |
| --- | --- | --- | --- | --- | --- | --- | --- | --- | --- | --- | --- | --- | --- |
| Welding activity | No. | No. | OR1^b^ | OR2^c^ | 95% CI | OR3^d^ | 95% CI | No. | OR3^d^ | 95% CI | No. | OR3^d^ | 95% CI |
| Reference group^a^ | 449 | 170 | 1.00 | 1.00 |  | 1.00 |  | 104 | 1.00 |  | 66 | 1.00 |  |
|  |  |  |  |  |  |  |  |  |  |  |  |  |  |
| Ever welding | 206 | 80 | 1.03 | 1.13 | 0.82-1.56 | 1.15 | 0.74-1.79 | 50 | 1.16 | 0.68-1.99 | 30 | 1.10 | 0.56-2.18 |
| Occasional | 193 | 70 | 0.96 | 1.06 | 0.76-1.48 | 1.09 | 0.69-1.70 | 45 | 1.12 | 0.65-1.94 | 25 | 1.02 | 0.51-2.05 |
| Regular | 13 | 10 | 2.03 | 2.18 | 0.91-5.25 | 2.55 | 0.96-6.82 | 5 | 1.92 | 0.58-6.37 | 5 | 3.46 | 0.91-13.1 |
|  |  |  |  |  |  |  |  |  |  |  |  |  |  |
| Cumulative exposure^e,f^ |  |  | 1.00 | 1.02 | 0.97-1.07 | 1.01 | 0.95-1.09 |  | 1.03 | 0.94-1.12 |  | 1.01 | 0.91-1.12 |
| Q1 and Q2 (0-830) | 108 | 44 | 1.08 | 1.14 | 0.77-1.71 | 1.12 | 0.70-1.79 | 27 | 1.10 | 0.62-1.95 | 17 | 1.10 | 0.53-2.28 |
| Q3 and Q4 (831-39,704) | 98 | 36 | 0.97 | 1.11 | 0.71-1.72 | 1.24 | 0.70-2.21 | 23 | 1.37 | 0.67-2.78 | 13 | 1.10 | 0.44-2.73 |
| T1 (0-431) | 69 | 32 | 1.23 | 1.30 | 0.81-2.07 | 1.17 | 0.69-2.00 | 20 | 1.21 | 0.64-2.30 | 12 | 1.09 | 0.49-2.42 |
| T2 (432-1,456) | 71 | 29 | 1.08 | 1.17 | 0.72-1.90 | 1.24 | 0.70-2.22 | 18 | 1.18 | 0.58-2.40 | 11 | 1.25 | 0.51-3.03 |
| T3 (1,457-39,704) | 66 | 19 | 0.76 | 0.88 | 0.50-1.55 | 0.91 | 0.45-1.83 | 12 | 0.95 | 0.40-2.27 | 7 | 0.87 | 0.30-2.53 |
|  |  |  |  |  |  |  |  |  |  |  |  |  |  |
| Duration of welding^e^, years |  |  | 1.01 | 1.06 | 0.92-1.21 | 1.07 | 0.88-1.29 |  | 1.10 | 0.89-1.39 |  | 0.99 | 0.73-1.34 |
| Q1 and Q2 (0-8) | 105 | 43 | 1.08 | 1.21 | 0.81-1.79 | 1.24 | 0.75-2.04 | 27 | 1.31 | 0.71-2.39 | 16 | 1.10 | 0.51-2.38 |
| Q3 and Q4 (9-49) | 101 | 37 | 0.97 | 1.08 | 0.72-1.62 | 1.04 | 0.60-1.79 | 23 | 0.99 | 0.51-1.93 | 14 | 1.10 | 0.48-2.51 |
| T1 (1-4) | 60 | 20 | 0.88 | 0.96 | 0.55-1.67 | 1.04 | 0.56-1.93 | 10 | 0.88 | 0.39-1.95 | 10 | 1.20 | 0.50-2.88 |
| T2 (5-12) | 79 | 37 | 1.24 | 1.30 | 0.83-2.01 | 1.29 | 0.75-2.21 | 23 | 1.33 | 0.69-2.58 | 14 | 1.22 | 0.54-2.73 |
| T3 (13-49) | 67 | 23 | 0.91 | 1.06 | 0.62-1.81 | 1.06 | 0.56-2.02 | 17 | 1.26 | 0.60-2.63 | 6 | 0.73 | 0.25-2.19 |
|  |  |  |  |  |  |  |  |  |  |  |  |  |  |
| Average intensity^e,g^ |  |  | 0.83 | 0.84 | 0.61-1.15 | 1.06 | 0.74-1.51 |  | 1.02 | 0.67-1.57 |  | 1.12 | 0.65-1.92 |
| Q1 and Q2 (0-92) | 102 | 43 | 1.11 | 1.22 | 0.81-1.84 | 1.18 | 0.73-1.92 | 26 | 1.13 | 0.63-2.05 | 17 | 1.19 | 0.57-2.47 |
| Q3 and Q4 (93-1,038) | 104 | 37 | 0.94 | 1.03 | 0.67-1.59 | 1.09 | 0.62-1.93 | 24 | 1.21 | 0.61-2.42 | 13 | 0.95 | 0.40-2.29 |
| T1 (0-64) | 64 | 27 | 1.11 | 1.14 | 0.70-1.88 | 1.08 | 0.62-1.88 | 18 | 1.12 | 0.58-2.15 | 9 | 0.95 | 0.40-2.28 |
| T2 (65-142) | 73 | 33 | 1.19 | 1.37 | 0.86-2.18 | 1.37 | 0.77-2.43 | 20 | 1.38 | 0.67-2.82 | 13 | 1.30 | 0.55-3.07 |
| T3 (143-1,038) | 69 | 20 | 0.77 | 0.86 | 0.50-1.49 | 0.98 | 0.51-1.91 | 12 | 0.98 | 0.43-2.24 | 8 | 1.10 | 0.41-2.96 |

Abbreviations: CI, confidence interval; OR, odds ratio Q, quartile; T, tertile.
a: Subjects who were never exposed-welding fumes.
b: Crude OR without any adjustment.
c: OR conditional on study area and 5-year age group.
d: OR additional adjusted for specific risk factors: History of cryptorchidism, suffering from testicular cancer of the father / brother, and ever exposure to metal-working fluids.
e: OR were computed by entering the log-transformed continuous variable (cumulative exposure+1; duration+1; average intensity+1) into the model.
f: Cumulative exposure to welding fumes presented in μg/m^3^-years.
g: Average intensity of exposure to welding fumes presented in μg/m^3^.
